# Severe Acute Chest Syndrome in a Sickle Cell Patient That Required Repeated Exchange Transfusion

**DOI:** 10.7759/cureus.13066

**Published:** 2021-02-01

**Authors:** Mohammed Saleh, Joseph Asemota

**Affiliations:** 1 Internal Medicine, University of Missouri, Columbia, USA; 2 Internal Medicine, Howard University Hospital, Washington, D.C., USA; 3 Clinical Anatomy, St. George's University School of Medicine, True Blue, GRD; 4 Hematology/Oncology, Beth Israel Deaconess Medical Center, Harvard Medical School, Boston, USA

**Keywords:** sickle cell disease, acute chest syndrome, exchange transfusion, packed red blood cell transfusion, severe respiratory failure

## Abstract

Acute chest syndrome (ACS) is one of the major causes of mortality and morbidity in patients with sickle cell disease (SCD). ACS usually presents in a more severe form in adults older than 20 years. High clinical suspicion should be maintained in SCD patients who presents with painful crises. This case report presents an interesting severe form of ACS that, quite unusually, required repeated exchanged blood transfusion to achieve clinical improvement.

## Introduction

Acute chest syndrome (ACS) is the most common acute disorder of the respiratory system in patients with sickle cell disease (SCD) [1]. It is a rapidly progressive illness characterized by new radio-density on chest radiograph, which is accompanied by fever and/or respiratory symptoms, with severe hypoxia being a useful predictor of severity and outcome [2].

Although ACS is more commonly seen in patients with hemoglobin SS (HbSS), it can occur in any other SCD phenotypes. In fact, about 50% of all patients with SCD will have an episode of ACS in their lifetime [1]. ACS is a major cause of morbidity and mortality in patients with SCD, and it has a peak incidence in the pediatric age group of two to four years [3]. Amongst adults, about 78% of ACS episodes are caused by vaso-occlusive pain crisis [3]. It accounts for about one in every four deaths in patients with SCD, with an estimated death rate of about 1.1% and 4.3% in the pediatric and adult populations, respectively [3].

Given that ACS usually presents in a severe form in adults, especially those older than 20 years, prompt and often aggressive intervention is particularly essential in preventing clinical deterioration in this sub-population. One of the mainstays of therapeutic interventions done in ACS is blood transfusion. Both simple and exchange blood transfusions have an important role in the management of severe cases of ACS, with guidelines recommending early simple blood transfusions with the option for exchange blood transfusion in the event of non-resolution after initial simple blood transfusion [3-5].

In this clinical management spectrum, most patients that require exchange transfusion often achieve resolution upon a single exchange transfusion. However, this report presents a unique case of a patient with a severe form of ACS that required repeated exchange blood transfusions to achieve clinical improvement.

## Case presentation

**Figure 1:**
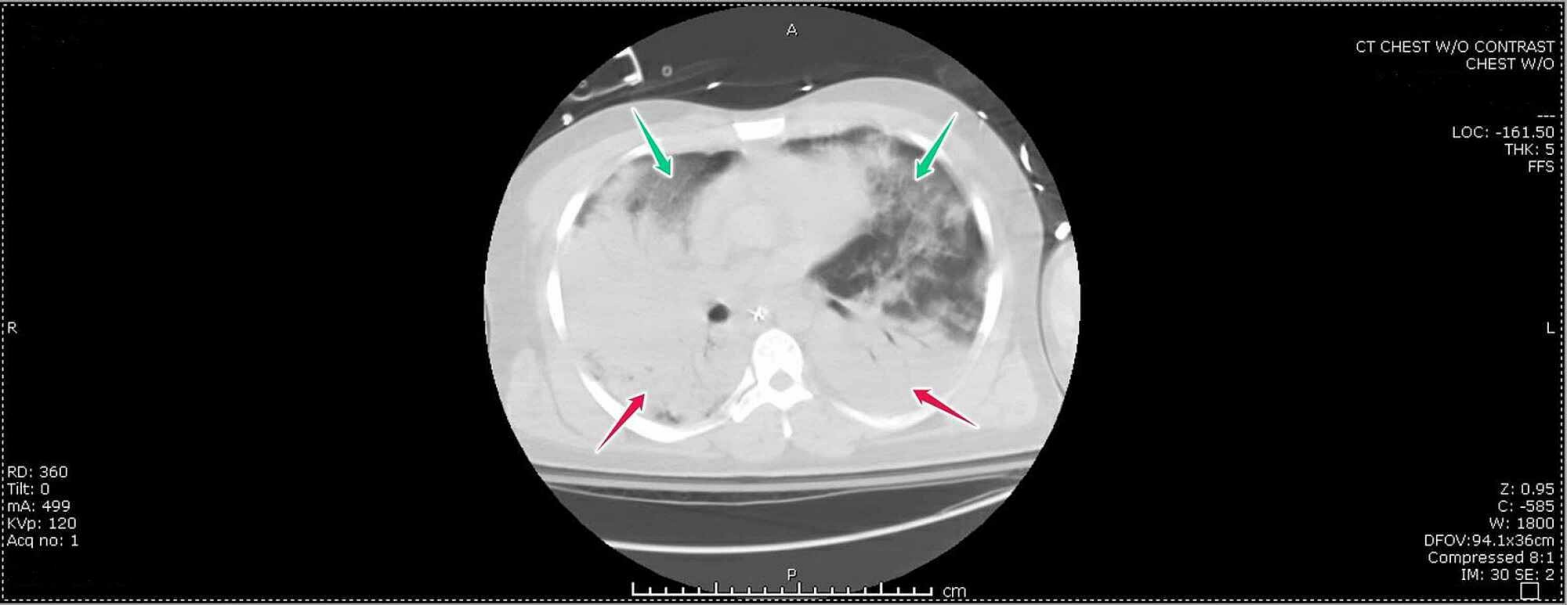
CT scan of the chest showing dense bilateral opacification of the mid and lower lung segments (red arrows) and ground-glass opacities in mid and upper segments bilaterally (green arrows).

A 35-year-old African American male with a medical history significant for asthma, active smoking (12-pack-year smoking history), pulmonary hypertension, and sickle cell disease presented to the hospital with complaints of productive cough and chest discomfort of five days duration. He also provided a history of associated fever and anorexia. Physical exam revealed tachycardia, pallor, and decreased airway entry in the right lung base. He had a baseline hemoglobin level of 8-9 g/dl. Investigations showed leukocytosis, reticulocytosis, and severe anemia. Chest X-ray showed bilateral infiltrates and a small right-sided pleural effusion. Shortly after admission, the patient’s condition deteriorated, and he developed acute respiratory distress. Arterial blood gas (ABG) PO2 was 58 mmHg on room air. Pan-cultures were obtained, and he was managed with oxygen, intravenous fluids (IVF), and empiric broad-spectrum intravenous antibiotics. His condition worsened, and repeat ABG while on 100% oxygen showed PO2 of 35 mmHg, after which he was transferred to the Medical Intensive Care Unit (MICU) and subsequently intubated for hypoxic respiratory failure. In the MICU, the patient’s hospital course progressively worsened with the development of adult respiratory distress syndrome. Computed tomography (CT) of the chest was done after intubation, and it showed opacification of the lung parenchyma most prominently in the mid to lower lung fields posteriorly (Figure 1).

The primary managing team initially transfused the patient with two units of packed red blood cells without significant clinical response. Due to the patient’s non-response, the hematology team was consulted, and their recommendation was to initiate an exchange blood transfusion. The patient received one complete course of exchange blood transfusion with no significant change in his clinical condition. His pan-cultures remained negative. With the continued deterioration of his clinical condition, the decision was made to initiate a second course of exchange blood transfusion. Upon completing the second course of exchange transfusion, his clinical condition markedly improved, and he remained stable. He was ultimately discharged home to follow up with the outpatient SCD clinic.

## Discussion

In patients with SCD, ACS can be present on admission or develop shortly after admission, i.e., within 24-72 hours [6]. Management should focus on pain relief, IVF hydration, maintaining oxygen above 94%, and blood transfusion [7-9].

In terms of transfusion, simple transfusion is indicated for mild disease while exchange blood transfusion (EBT) is often reserved for patients with severe forms of disease that do not respond to simple transfusion, with evidence of severe hypoxemia and multilobar disease on chest radiograph. Eight units of packed red blood cells (PRBC) of exchange transfusion is usually sufficient to lower the sickle cell proportion and see clinical improvement. Specifically, the goal is to decrease the proportion of circulating HbS to <30% while simultaneously increasing the hemoglobin concentration to approximately 10 g/dL [2].

Exchange transfusion, therefore, effectively reduces the number of sickle cells in circulation and prevents their participation in vaso-occlusive events with the unique benefit of increasing hematocrit without a concomitant rise in blood viscosity [10].

In our patient, despite receiving one session of exchange blood transfusion, his clinical condition continued to deteriorate and prognosis seemed poor. The exact cause for the non-response to initial exchange transfusion remains unclear, but it is opined that the patient’s smoking habit and his relatively higher baseline hemoglobin levels placed him at risk for a more severe form of ACS. Also, other genetic factors such as higher baseline levels of P-selectin could have contributed to the patient’s clinical course [11]. 

Although the team was initially hesitant to repeat exchange transfusion given the potential risks associated with transfusions, the exchange transfusion was ultimately repeated with significant improvement in the patient’s clinical course. This case clearly underscores the need to consider repeat exchange transfusion in the management of ACS if there is no improvement in the clinical course after an initial session of exchange blood transfusion.

## Conclusions

While guidelines provide a broad framework for the management of patients with ACS, they do not specifically provide a protocol for non-response to exchange transfusion. The decision to repeat exchange transfusion and how soon after a previous exchange transfusion is, therefore, left to the clinical judgment of the practitioner. Our patient presented with severe ACS that was initially unresponsive to exchange transfusion. The details of the case highlight the importance of repeating exchange transfusion until recovery is achieved and maintained.
